# Altitudinal Distribution Patterns of Phyllosphere Microbial Communities and Their Contribution to Silage Fermentation of *Kobresia pygmaea* Along the Elevation Gradient on the Tibetan Plateau

**DOI:** 10.3389/fmicb.2022.874582

**Published:** 2022-05-24

**Authors:** Xin Yang, Yuhong Bao, Tao Shao, Wenkang Wang, Pengfei Ma, Wenbo Wang, Antonio Gallo, Xianjun Yuan

**Affiliations:** ^1^Institute of Ensiling and Processing of Grass, College of Agro-Grassland Science, Nanjing Agricultural University, Nanjing, China; ^2^State Key Laboratory of Germplasm Resources and Genetic Improvement of Tibetan Barley and Yak, Lhasa, China; ^3^Institute of Grass Science, TAR Academy of Agricultural and Animal Husbandry Sciences, Lhasa, China; ^4^Department of Animal Science, Food and Nutrition (DIANA), Faculty of Agricultural, Food and Environmental Sciences, Università Cattolica del Sacro Cuore, Piacenza, Italy

**Keywords:** elevational gradients, *Kobresia pygmaea*, fermentation quality, Tibetan Plateau, bacterial diversity, fungal diversity

## Abstract

The study aimed to reveal altitudinal distribution patterns of phyllosphere microbial communities and silage fermentation of *Kobresia pygmaea* along the elevation gradient on the Tibetan Plateau. The *K. pygmaea* was individually collected from 2,500, 3,000, 4,000, 4,500, and 5,000 m above sea level (a.s.l.) on the Tibetan Plateau and ensiled for 60 days, respectively. The phyllosphere bacterial diversity increased while fungal diversity decreased along the elevation gradient, and bacterial and fungal richness showed a unimodal distribution with peak abundance at 4,000 and 3,000 m a.s.l., respectively. After 60 days of ensiling, the bacterial and fungal community composition changed but did not exhibit clear altitudinal distribution patterns. All *K. pygmaea* underwent a weak fermentation indicated by pH above 5.0 and low ratio of lactic/acetic acid (LA/AA). The S5000 and S3000 showed the highest and lowest pH, respectively. Although *Lactobacillus* dominated S4000 after 60 days of ensiling, S4000 still exhibited poor fermentation quality as well as silages from the other four regions. The higher ammonia N concentrations in S3000 and S4000 than the other silages were consistent with the detectable butyric acid in S3000 and S4000. The silage fermentation of *K. pygmaea* collected from five regions exhibited poor fermentation quality, thereby inoculating lactic acid bacteria to *K. pygmaea* before ensiling is highly recommended to improve fermentation quality on the Tibetan Plateau.

## Introduction

The Tibetan Plateau, known as the “roof of the world,” has a unique ecosystem, which is characterized by extreme environmental conditions, including low temperature, strong ultraviolet rays, and hypoxia. The Tibetan Plateau harbors vast areas of alpine meadow; however, the forage yields are too low to feed the domestic animals, resulting in feed deficiency during the long cold period of a year (Zhou et al., 2017). Therefore, ensiling is a preferred forage preservation strategy to alleviate the seasonal unbalance of feed supply on the Tibetan Plateau. Ensiling fermentation is a complex biological process driven by epiphytic lactic acid bacteria (LAB) under anaerobic conditions, thus, the phyllosphere microbial flora played a critical role during ensiling (McAllister et al., 2018). It is well known that environmental factors affected the microbial community that played a significant role in ecosystem functioning and primary production (Handley, 2019). The Tibetan Plateau harbors unique microorganisms because it is geographically and ecologically distinct. Microbial abundance and species diversity vary considerably along environmental gradients across the plateau (Dong et al., 2010); however, the distribution patterns of phyllosphere microbial communities in *Kobresia pygmaea* along the elevation gradient on the Tibetan Plateau are still unclear.

Unraveling the distribution pattern of phyllosphere microbial communities on the Tibetan Plateau has been attracting the growing scientific interests of ecologists. The phyllosphere microbiome has been shown to play an important role in the adaptation of the plant host to different environmental stressors by enhancing tolerance to heat, cold, drought, and salinity (Al Ashhab et al., 2021). Investigating the change in bacterial communities along the altitudinal gradient will shed light on the prediction of future climatic changes scenario (Yuan et al., 2014). Wei et al. (2022) characterized the microbial community composition and metabolic capacity in Qinghai-Tibet Plateau high-altitude areas (with an altitude of 4,555 m) and confirmed that Qinghai-Tibet Plateau is a huge and valuable resource bank in which more new non-resistant antibiotics and many other bioactive substances could be developed. Characterizing the microbial community composition is important for forage preservation because phyllosphere microbial flora play a critical role in ensiling fermentation and provide sufficient fodder for local livestock during winter and spring. Thus, surveys on phyllosphere microbial community composition will reveal the key and dominant microorganism in grass along altitudinal gradients, which might be useful as a potential application in silage fermentation in the different regions of the Tibetan Plateau.

*Kobresia pygmaea* is an important forage resource in the Qinghai-Tibet Plateau and is essential in maintaining the ecological balance of grasslands. *K. pygmaea* is widely distributed in temperate to cold zones of the Himalayas and Hengduan Mountains because they are tolerant to cold, radiation, drought, and strong wind (Can et al., 2020). Ding et al. (2020) proposed that the harsh and unique environment contributed to the unique epiphytic microbial community of forages on the Tibetan Plateau compared to other regions worldwide, which subsequently influenced silage fermentation quality of native grasses.

The study aimed to reveal the altitudinal distribution of phyllosphere microbial community and evaluate their contributions to silage fermentation of *K. pygmaea* harvested from five elevation gradients on the Tibetan Plateau. We hypothesized that (1) the epiphytic fungal and bacterial communities in *K. pygmaea* varied along the elevation gradient on the Tibetan Plateau and (2) the variation in microbial communities might affect the silage fermentation of *K. pygmaea*.

## Materials and Methods

### Sampling Area and Silage Preparation

*Kobresia pygmaea* was individually collected from 2,500, 3,000, 4,000, 4,500, and 5,000 m above sea level (a.s.l.) on the Tibetan Plateau. The exact coordinates and climatic characteristics of the sampling sites are shown in Supplementary Table S1. For each elevation gradient, five plots (100 × 50 m block) with vegetation coverage of *K. pygmaea* above 80% were chosen for parallel sampling, and the grass plots were open terrain and far away from residential areas. Five squares (50 × 50 cm) per plot were chosen for fresh *K. pygmaea* collecting. All collected fresh forage were mixed and chopped into lengths of 2–3 cm with a paper cutter. Then, about 500 g of chopped grasses were packed into polyethylene plastic bags (dimensions 270 × 300 mm) and vacuum-sealed tightly, and five bags for each elevation gradient were prepared. All bags were taken to the laboratory in Lhasa and stored at ambient temperature (20–25°C) for 60 days. At the same time, the fresh forages in quintuplicate per elevation gradient were taken for immediate microbial population analyses and DNA extraction, and a portion of fresh forages (80 g) was sampled for dry matter (DM) and chemical composition analyses.

### Laboratory Analyses

Five bags per elevation gradient were opened after 60 days of ensiling, then all silages of each bag were poured into an ethanol-sterilized plastic container and mixed thoroughly. One subsample (80 g) of fresh *K. pygmaea* and silage was dried in a forced-air oven at 65°C for 48 h for the determination of DM. Then, dried samples were ground in a laboratory knife grinder (FW100, Taisite Instrument Co., Ltd, Tianjin, China) to pass a 1-mm sieve. Ground samples were used for total nitrogen (TN), crude protein (CP), water-soluble carbohydrate (WSC), neutral detergent fiber (NDF), and acid detergent fiber (ADF) analyses. The TN content was determined with a Kjeltec Analyzer (Kjeltec 8400 -Analyzer, FOSS Analytical AB, Höganäs, Sweden), and the CP content was calculated by multiplying the TN by 6.25 (Krishnamoorthy et al., 1982). The WSC content was analyzed by colorimetry after reacting with the Throne reagent (Thomas, 1977). An ANKOM 200 fiber analyzer (ANKOM Technologies, Macedon, NY, USA) was used to determine the NDF and ADF according to the Van Soest procedures, a thermostable α-amylase was used, and the results were expressed on a DM basis, including residual ash.

A subsample of silage (20 g wet basis) was diluted with 60 ml distilled water and stored at 4°C for 24 h. The silage extract was filtered through four layers of cheesecloth and Whatman filter paper (Hangzhou Xinhua Co., Ltd., China). The pH of the extract was measured immediately using a glass electrode pH meter (Hanna Instruments Italia Srl, Padua, Italy). Ammonia N was measured according to the phenol-hypochlorite method (Broderick and Kang, 1980). One subsample (5 ml) of silage extract was centrifuged at 12,000 × *g* for 10 min at 4°C, and the supernatant was filtered through a 0.45-μm membrane for organic acid analysis. Organic acids were quantified using a high-performance liquid chromatography system (Agilent HPLC 1260, Agilent Technologies, Inc., Waldbronn, Germany) equipped with a refractive index detector.

A subsample of fresh or silage (10 g) was mixed and homogenized with 90 ml of sterile sodium chloride solution (0.85%) for 1 min. The homogenate was filtered through four layers of cheesecloth, then 1 ml of the filtrate was serially diluted for microorganism counting, and the remaining solution was filtered through four layers of medical gauze for DNA extraction. The numbers of LAB were counted on deMan, Rogosa, and Sharp agar after 48 h of incubation at 37°C under anaerobic conditions. The Enterobacteriaceae were counted on purple-red bile glucose agar after 24 h of incubation at 37°C under aerobic conditions. The numbers of yeast and molds were counted on potato dextrose agar after culturing for 48–72 h under aerobic conditions at 28°C. The numbers of aerobic bacteria were counted on the nutrient agar medium (Qingdao Haibo Biotechnology Co., Ltd) after aerobic incubation for 2 days at 37°C. Microbial data was obtained in the form of colony-forming units (CFU) and converted to a logarithmic scale on a fresh weight (FW) basis.

### Microbial Community Analyses

Three replicates of fresh or silage per elevation gradient were randomly chosen for microbial community analyses by sequencing the bacterial 16S rRNA V3–V4 region and the fungal internal transcribed spacer (ITS) region. The DNA extraction solution was centrifuged at 10,000 × *g* for 15 min, then the supernatant was discarded, and the pellet was resuspended by 100 μl of autoclaved Ringer's solution. Microbial DNA was extracted using the Fast DNA SPIN Kit for Soil (MP Biomedicals, Solon, OH, USA) following the protocol provided by the manufacturer. The quantity and quality of DNA were evaluated using NanoDrop 2000 ultraviolet-visible spectrophotometer (Thermo Scientific, Wilmington, DE, USA), and DNA quality was checked by 1% agarose gel electrophoresis. The bacterial 16S rRNA V3–V4 regions were amplified based on the specific primers 338F (ACTCCTACGGGAGGCAGCAG) and 806R (GGACTACHVGGGTWTCTAAT). The primers ITS1F (5′-CTTGGTCATTTAGAGGAAGTAA-3′) and ITS2aR (5′-GCTGCGTTCTTCATCGATGC-3′) were used to amplify fungal ITS. The purified PCR amplicons were paired-end sequenced using the Illumina MiSeq PE300 platform (Illumina Inc., San Diego, CA, USA). Sequences have been deposited in the NCBI Short Read Archive database under BioProject PRJNA742870.

The sequence was first quality-controlled to remove mismatches and ambiguous readings. Then, operational taxonomic units (OTUs) were clustered with a 97% similarity cutoff using UPARSE (version 7.1). Bacterial community structure was analyzed at the phylum and genus levels using the Silva database (release 138) with a confidence threshold of 70%, respectively. Fungal community structure was analyzed at the phylum and genus levels using the Unite 8.0 database (release 8.0) with a confidence threshold of 70%, respectively. Alpha-diversity estimates and beta-diversity evaluation, based on principal coordinate analysis (PCoA), were performed using the Phyloseq and Vegan packages on R. The distance-based redundancy analysis was used to examine the relationship between bacterial communities and environmental factors, whereas canonical correspondence analysis (CCA) was used to examine the relationship between fungal communities and environmental factors with Canoco for Windows 4.5 (Microcomputer Power, Ithaca, NY, USA).

### Statistical Analysis

The chemical composition and microbial populations of fresh *K. pygmaea* and silages were analyzed as a completely randomized design using the GLM procedure of SAS (version 9.3; SAS Institute Inc., Cary, NC) with a model that included the elevation gradient effect. A polynomial contrast was used to test the linear or quadratic effects of elevation gradient on parameters measured. The data of bacterial and fungal diversity were analyzed as a completely randomized factorial design in a 2 (fresh and silage) × 5 (elevation gradients) factorial arrangement, with the main effect of elevation gradient, ensiling, and their interactions by the GLM procedure of SAS. If significance was detected for a specific effect or interaction (*p* ≤ 0.05), data were analyzed using Tukey's test.

## Results

### Chemical Composition and Microbial Populations in Fresh *K. pygmaea*

The chemical composition and microbial populations of fresh *K. pygmaea* along the elevation gradient on the Tibetan Plateau are shown in Table 1. The DM contents of fresh *K. pygmaea* ranged from 283 to 561 g kg^−1^, and the elevation gradient had a significant influence on DM contents with a linear and quadratic effect (*p* < 0.01). The NDF (*p* = 0.358) and ADF (*p* = 0.193) contents were similar for all fresh *K. pygmaea*. The elevation gradient significantly affected CP content with a linear and quadratic effect (*p* < 0.01). The elevation gradient significantly affected WSC content with a linear effect (*p* < 0.01). The numbers of LAB, yeast and molds were affected by elevation gradient with a quadratic effect (*p* = 0.011). The elevation gradient significantly affected the numbers of Enterobacteriaceae and aerobic bacteria with a linear and quadratic effect (*p* < 0.01).

**Table 1:** The chemical composition (g kg^−1^ DM basis unless stated otherwise) and microbial populations (log _10_ CFU g^−1^ FW) of fresh *K. pygmaea* along the elevation gradient on the Tibetan Plateau.

**Item**	**Altitude^1^**					**SEM^2^**	***P*-value**	**Contrast** ***P*****-values**	
	**F2500**	**F3000**	**F4000**	**F4500**	**F5000**			**Linear**	**Quadratic**
Dry matter (g kg^−1^ FW)	295^d^	283^d^	315^c^	359^b^	561^a^	27.4	<0.01	<0.01	<0.01
Neutral detergent fiber	422	450	455	460	463	6.9	0.358	0.077	0.381
Acid detergent fiber	201	238	214	221	220	4.9	0.193	0.484	0.241
Crude protein	108^c^	128^b^	158^a^	128^b^	131^b^	4.4	<0.01	<0.01	<0.01
Water-soluble carbohydrate	16.3^a^	10.7^b^	14.3^a^	9.83^b^	9.02^b^	0.796	<0.01	<0.01	0.555
Lactic acid bacteria	3.00^b^	5.03^a^	3.40^b^	3.77^ab^	3.00^b^	0.232	<0.01	0.228	0.011
Yeast and Molds	2.30^ab^	3.31^a^	2.79^ab^	2.40^ab^	2.00^b^	0.149	0.019	0.065	0.011
Enterobacteriaceae	5.26^b^	6.66^a^	5.24^b^	5.26^b^	4.00^c^	0.229	<0.01	<0.01	<0.01
Aerobic bacteria	5.80^b^	6.66^a^	6.13^ab^	5.41^b^	4.00^c^	0.248	<0.01	<0.01	<0.01

a−d *Values within the same row with different letters are significantly different (p < 0.05)*.

1 *F2500, F3000, F4000, F4500, and F5000 represent fresh K. pygmaea at corresponding altitude gradients*.

2 *SEM, standard error of the mean*.

### Chemical Composition, Microbial Populations, and Fermentation Quality of *K. pygmaea* Silages After 60 Days of Ensiling

The chemical composition, microbial population, and fermentation profiles of *K. pygmaea* silage along the elevation gradient are shown in Tables 2, 3. Quadratic effect (*p* < 0.01) of elevation gradient on DM and CP contents was detected. The elevation gradient significantly affected contents of ADF with linear (*p* < 0.01) effect. The WSC contents in silage linearly (*p* < 0.01) increased with increasing elevation gradient. There was a quadratic effect (*p* < 0.01) of elevation gradient on the numbers of Enterobacteriaceae and aerobic bacteria. The LAB numbers in silages linearly (*p* < 0.01) declined with the increasing elevation gradient. The silage pH linearly (*p* < 0.01) increased with the increasing elevation gradient. The elevation gradient affected (*p* < 0.01) the LA concentration with the highest LA values in S3000. The AA concentration exhibited the quadratic (*p* < 0.01) effect with the lowest AA concentration in S4500. A similar PA concentration was observed in S2500, S3000, and S4000 silage, and it was not detected in S4500 and S5000 silages. The BA was solely detected in S3000 and S4000 silages. Linear and quadratic effects (*p* < 0.01) of elevation gradient on ammonia N concentration were detected.

**Table 2:** The chemical composition (g kg^−1^ DM basis unless stated otherwise) and microbial populations (log _10_ CFU g^−1^ FW) of ensiled *K. pygmaea* along the elevation gradient on the Tibetan Plateau.

**Item**	**Altitude^1^**					**SEM^2^**	***P*-value**	**Contrast** ***P*****-values**	
	**S2500**	**S3000**	**S4000**	**S4500**	**S5000**			**Linear**	**Quadratic**
Dry matter (g kg^−1^ FW)	280^c^	278^c^	261^d^	351^b^	567^a^	33.0	<0.01	<0.01	<0.01
Neutral detergent fiber	453^ab^	480^a^	389^b^	443^ab^	439^ab^	9.6	0.015	0.194	0.157
Acid detergent fiber	270^a^	261^a^	185^b^	209^b^	196^b^	9.8	<0.01	<0.01	0.016
Crude protein	107^c^	116^c^	143^a^	131^b^	142^ab^	3.9	<0.01	<0.01	<0.01
Water-soluble carbohydrate	5.56^b^	5.41^b^	5.53^b^	5.79^b^	7.13^a^	0.176	<0.01	<0.01	<0.01
Lactic acid bacteria	7.49^a^	5.00^c^	6.91^b^	5.39^c^	5.00^c^	0.282	<0.01	<0.01	0.105
Yeast and molds	2.26	3.09	2.55	2.42	3.55	0.202	0.234	0.174	0.530
Enterobacteriaceae	5.18^ab^	3.00^c^	3.50^bc^	5.21^ab^	5.41^a^	0.303	<0.01	0.046	<0.01
Aerobic bacteria	6.41^ab^	5.75^bc^	5.04^c^	6.27^ab^	7.00^a^	0.198	<0.01	0.047	<0.01

a−d *Values within the same row with different letters are significantly different (p < 0.05)*.

1 *S2500, S3000, S4000, S4500, and S5000 represent K. pygmaea silage after 60 days of ensiling at corresponding altitude gradients*.

2 *SEM, standard error of the mean*.

**Table 3:** Fermentation profiles of *K. pygmaea* silage along the elevation gradient on the Tibetan Plateau.

**Item**	**Altitude^1^**					**SEM^2^**	***P*-value**	**Contrast** ***P*****-values**	
	**S2500**	**S3000**	**S4000**	**S4500**	**S5000**			**Linear**	**Quadratic**
pH	5.25^bc^	5.24^c^	5.40^bc^	5.72^ab^	5.75^a^	0.055	<0.01	<0.01	0.070
Lactic acid (g kg^−1^ DM)	26.3^bc^	42.1^a^	18.9^cd^	30.6^b^	16.3^d^	2.57	<0.01	<0.01	<0.01
Acetic acid (g kg^−1^ DM)	24.8^ab^	20.0^bc^	19.7^bc^	14.0^c^	29.3^a^	1.55	<0.01	0.605	<0.01
Lactic/acetic acid	1.08^bc^	2.15^ab^	0.98^c^	2.29^a^	0.56^c^	0.204	<0.01	0.256	<0.01
Propionic acid (g kg^−1^ DM)	8.99	8.28	8.60	0.00	0.00	1.485	0.051	<0.01	0.449
Butyric acid (g kg^−1^ DM)	0.00^b^	14.3^a^	2.88^b^	0.00^b^	0.00^b^	1.563	<0.01	0.512	<0.01
Ammonia N (g kg^−1^ TN)	33.6^c^	78.9^a^	51.2^b^	26.4^c^	11.4^d^	6.25	<0.01	<0.01	<0.01

a−d *Values within the same row with different letters are significantly different (p < 0.05)*.

1 *S2500, S3000, S4000, S4500, and S5000 represent K. pygmaea silage after 60 days of ensiling at corresponding altitude gradients*.

2 *SEM, standard error of the mean*.

### Bacterial and Fungal Communities of Fresh and Ensiled *K. pygmaea* Along the Elevation Gradient on the Tibetan Plateau

Sequencing bacterial 16S rRNA V3–V4 and fungal ITS regions resulted in 1,747,180 and 2,001,636 reads, respectively. The Good's coverage values were greater than 99% for all samples, indicating that the depth of sequencing was adequate for reliable analysis of the bacterial and fungal communities. Rarefaction curves plateaued in all samples, indicating that the number of reads used in the analysis was sufficient for identifying OTU. The α-diversity indexes of the bacterial and fungal communities of fresh and ensiled *K. pygmaea* are shown in Table 4. There was an interaction between elevation gradient and ensiling for bacterial Shannon (*p* < 0.01) and Chao 1 (*p* = 0.018) indexes. The bacterial Shannon index was similar among all fresh grasses, whereas S5000 showed the lowest bacterial Shannon index among all silages. The Chao 1 index in F4000 was the highest among all fresh grasses, whereas S5000 showed the lowest Chao 1 index among silages. There was an interaction (*p* < 0.01) between elevation gradient and ensiling for fungal Shannon (*p* < 0.01) and Chao 1 (*p* = 0.017) indexes. F5000 showed the lowest fungal Shannon index among fresh grasses, whereas there was no significant difference in Shannon index among all silages. The fungal Chao 1 index in F3000 was the highest among all fresh grasses, whereas in S4000 it was the highest among all silages.

**Table 4:** The α-diversity indexes of the bacterial and fungal communities of *K. pygmaea* along the elevation gradient on the Tibetan Plateau.

**Item**	**Bacterial community**				**Fungal community**			
	**Shannon**		**Chao 1**		**Shannon**		**Chao 1**	
**Altitudes**	**Fresh**	**Silage**	**Fresh**	**Silage**	**Fresh**	**Silage**	**Fresh**	**Silage**
2,500	2.75	2.89^a^	416^b^	223^ab^	3.36^a^	2.74	228^bc^	359^ab^
3,000	2.81	2.59^a^	482^ab^	262^a^	3.64^a^	1.45	460^a^	393^ab^
4,000	3.19	2.34^ab^	659^a^	245^a^	2.44^ab^	3.46	332^ab^	444^a^
4,500	3.56	2.33^ab^	561^ab^	199^ab^	2.89^ab^	2.01	130^c^	323^b^
5,000	3.33	1.64^b^	340^b^	157^b^	1.20^b^	2.58	180^bc^	294^b^
***K. pygmaea***
Fresh	3.13^a^		492^a^		2.71^a^		266^b^	
Silage	2.35^b^		217^b^		2.45^b^		362^a^	
**Altitudes**
2,500	2.82		319^bc^		3.05		294^bc^	
3,000	2.70		372^ab^		2.55		426^a^	
4,000	2.77		452^a^		2.95		388^ab^	
4,500	2.94		380^ab^		2.45		226^c^	
5,000	2.49		248^c^		1.89		237^c^	
SEM^1^	0.110		31.4		0.193		29.3	
**Effects and interactions**
Elevation gradient	0.206		<0.01		0.168		<0.01	
Ensiling	<0.01		<0.01		0.409		<0.01	
Elevation gradient × Ensiling	<0.01		0.018		<0.01		0.017	

a−c *Values within the same column with different letters were significantly different (p < 0.05)*.

1 *SEM, standard error of the mean*.

**Figure 1:**
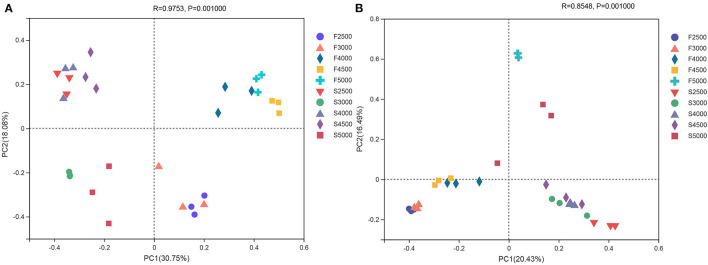
The unweighted principal coordinate analysis (PCoA) of bacterial **(A)** and fungal **(B)** community of *K. pygmaea* along the elevation gradient on the Tibetan Plateau. F2500, F3000, F4000, F4500, and F5000 represent samples of fresh *K. pygmaea* at corresponding altitude gradients. S2500, S3000, S4000, S4500, and S5000 represent samples of *K. pygmaea* silages at corresponding altitude gradients. Percentage variation explained by each PCoA is indicated on the axis.

The unweighted PCoA UniFrac plots revealed differences in the bacterial and fungal communities of fresh and ensiled *K. pygmaea* (Figure 1). For the bacterial community, principal component 1 (PC1) and principal component 2 (PC2) explained 30.75 and 18.08% of total variation, respectively (Figure 1A). The fresh grasses and silages were separated in the plot, and the fresh material harvested from low altitudes was separated from that of high altitudes. The PCoA plot revealed a clear separation and difference in the distribution and structure of the fungal communities between fresh and ensiled *K. pygmaea*, and the PC1 and PC2 explained 20.43 and 16.49% of total variation, respectively (Figure 1B). The fresh *K. pygmaea* were separated from silages. The fresh *K. pygmaea* and silages harvested from 5000 m were clustered far from samples harvested from other altitudes.

**Figure 2:**
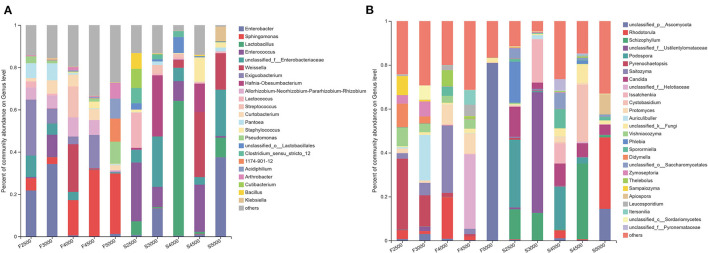
The bacterial **(A)** and fungal **(B)** community on genus levels of fresh and ensiled *K. pygmaea* along the elevation gradient on the Tibetan Plateau. F2500, F3000, F4000, F4500, and F5000 represent samples of fresh *K. pygmaea* at corresponding altitude gradients. S2500, S3000, S4000, S4500, and S5000 represent samples of *K. pygmaea* silages at corresponding altitude gradients.

A total of 23 bacterial genera with relative abundance (RA) above 5% were detected in samples (Figure 2A). *Enterobacter, Exiguobacterium*, and *Sphingomonas* were the dominant genera in F2500. *Enterobacter* was the most dominant genus in F3000. *Weissella* and *Sphingomonas* were the dominant genera in F4000. *Sphingomonas* was the most dominant genus in F5000 and F4500. After 60 days of ensiling, *Enterococcus, Lactococcus*, and *Lactobacillus* accounted for 28, 16.5, and 6.4% of the total sequence in S2500, respectively. *Hafnia-Obesumbacterium, unclassified_f_Enterobacteriaceae, Enterobacter*, and *Enterococcus* accounted for 29, 23.8, 13.3, and 9.6% of all sequences in S3000, respectively. *Lactobacillus* and *Enterococcus* accounted for 64 and 9.4% of the total sequence in S4000, respectively. *Weissella* and *Enterococcus* accounted for 31 and 22% of the total sequence in S4500, respectively. *Enterobacter, unclassified_f_Enterobacteriaceae, Weissella*, and *Lactobacillus* accounted for 37, 22, 17, and 9.3% of the total sequence in S5000.

For the fungal community, 27 fungal genera with RA above 5% were detected in all samples (Figure 2B). *Pyrenochaetopsis* and *Didymella* accounted for 32.3 and 10.8% of the total sequence in F2500, which decreased to 14.2 and 3.1% in F3000 silages, respectively. *Auriculibuller* became the most dominant genus in F3000. *Saitozyma* increased from 2.4% in F2500 to 30.6% of the total sequence for F4000. *Unclassified_f_Helotiaceae* and *Unclassified_p_Ascomycota* were the most dominant genera in F4500 and F5000, respectively. After 60 days of ensiling, *Podospora, Phlebia, Schizophyllum*, and *Candida* became the dominant genera in S2500. *Unclassified_f_Ustilentylomataceae, Issatchenkia*, and *Schizophyllum* were the dominant genera in S3000. S4000 had the most genera with RA above 5%, and *Podospora* was the most dominant genus. *Schizophyllum* and *Cystobasidium* were the dominant genera in S4500. *Rhodotorula* dominated the fungal community of S5000. *Candida* was present in all silages with the highest RA in S2500. The RA of *Schizophyllum* in S2500, S3000, and S4500 were above 10%. There was a quadratic trend in the RA of *Issatchenkia* along the elevation gradient, with the highest RA in S3000.

### Effect of Elevation Gradient and Abiotic Factors on the Microbial Communities of *K. pygmaea* Along the Elevation Gradient

**Figure 3:**
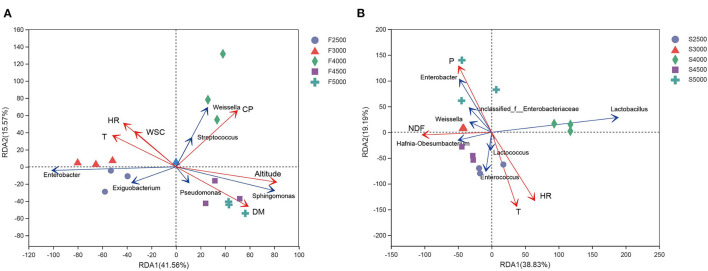
Redundancy analysis of environmental factors and bacterial community of fresh **(A)** and ensiled **(B)** *K. pygmaea* along the elevation gradient on the Tibetan Plateau. The axes are labeled with the percentage of total variance explained (%). Red arrow lengts indicate the variance explained by environmental factors, and the blue arrow lengths indicate the variance explained by the bacterial community. T Individual samples are represented with circles, triangles, diamonds, squares, and cross. F2500, F3000, F4000, F4500, and F5000 represent samples of fresh *K. pygmaea* at corresponding altitude gradients. S2500, S3000, S4000, S4500, and S5000 represent samples of *K. pygmaea* silages at corresponding altitude gradients. T, temperature; HR, relative humidity; WSC, water-soluble carbohydrate; CP, crude protein; DM, dry matter; P, average precipitation; NDF, neutral detergent fiber.

To analyze the effect of elevation gradient and abiotic factors on the microbial communities along the elevation gradient, RDA was performed for the bacterial communities of fresh and ensiled *K. pygmaea*. Altitude, temperature, WSC, CP, DM, and relative humidity had a significant effect on the variability of epiphytic bacterial communities and explained up to 57.13% (41.56 and 15.57% on RDA axes 1 and 2, respectively) of total variance of bacterial community (Figure 3A). In comparison, precipitation, relative humidity, temperature, and NDF had a significant effect on the bacterial communities of silages, explaining up to 58.02% (38.83 and 19.19% on RDA axes 1 and 2, respectively) (Figure 3B) of total variance of bacterial community of silages.

**Figure 4:**
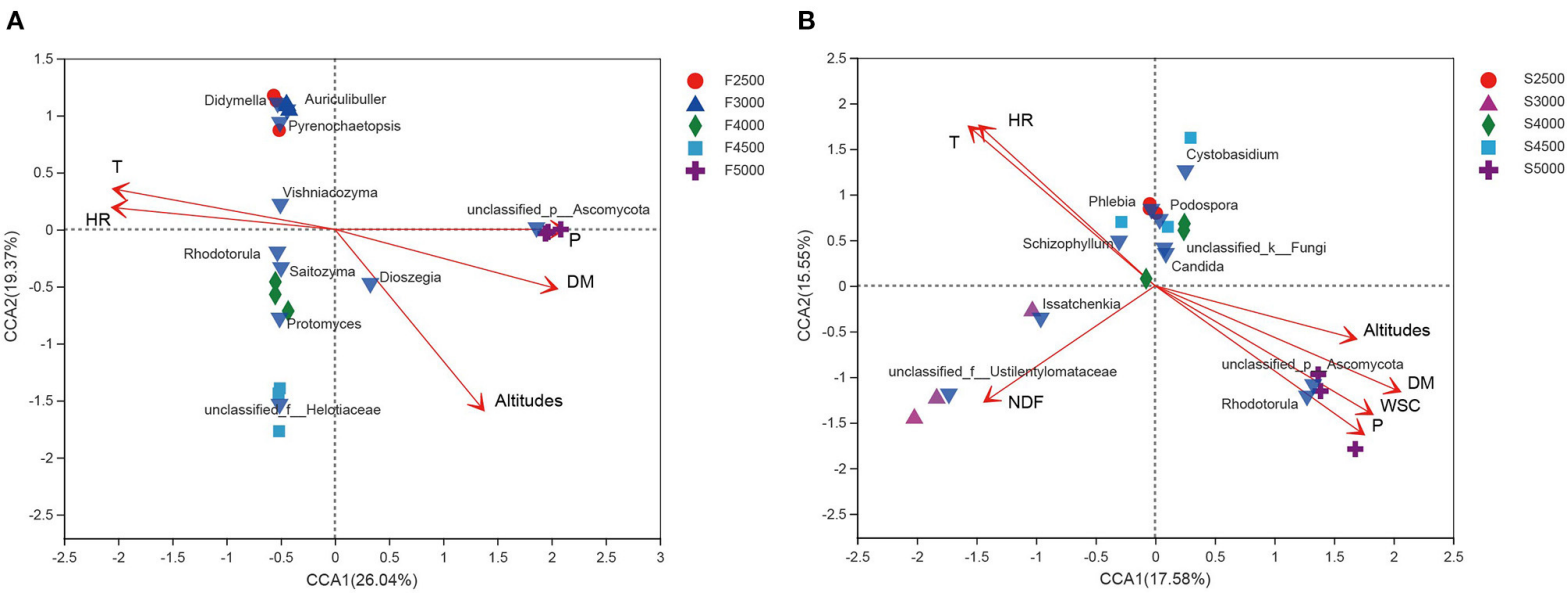
Canonical correspondence analysis of environmental factors and fungal community of fresh **(A)** and ensiled **(B)** *K. pygmaea* along the elevation gradient on the Tibetan Plateau. The canonical axes are labeled with the percentage of total variance explained (%). Red arrow lengths indicate the variance explained by environmental factors, and bright blue inverted triangle represents the interpretation of the fungal community presented as a single data point. Individual samples are represented with circles, triangles, diamonds, squares, and cross. F2500, F3000, F4000, F4500, and F5000 represent samples of fresh *K. pygmaea* at corresponding altitude gradients. S2500, S3000, S4000, S4500, and S5000 represent samples of *K. pygmaea* silages at corresponding altitude gradients. T, temperature; HR, relative humidity; WSC, water-soluble carbohydrate; DM, dry matter; P, average precipitation; NDF, neutral detergent fiber.

The CCA was performed for fungal community of fresh and ensiled *K. pygmaea*. For the fresh *K. pygmaea*, relative humidity, temperature, DM, elevation gradient, and precipitation had a significant effect on the variability of fungal community of fresh *K. pygmaea* and were explainable up to 45.41% (26.04 and 19.37% on CCA axes 1 and 2, respectively) of total variance of fungal community (Figure 4A). In comparison, relative humidity, temperature, NDF, DM, WSC, altitude, and precipitation had a significant effect on the fungal community of *K. pygmaea* silages, explaining up to 33.13% (17.58 and 15.55% on CCA axes 1 and 2, respectively) (Figure 4B) of total fungal community variability.

## Discussion

### Variations in Chemical Composition and Microbial Community of Fresh *K. pygmaea* Along the Elevation Gradient

In the Tibetan Plateau, alpine plants have developed special strategies to help survive the harsh environments, including sharp temperature shifts, strong ultraviolet radiation exposure, and low oxygen content (Li et al., 2014). The restrained plant respiration induced by decreasing temperature along the elevation gradients resulted in the accumulation of soluble carbohydrate, CP, and ether extract in cell protoplasm of the plant (Ding et al., 2020). In this study, the CP concentration of fresh *K. pygmaea* increased up to 15.76 g kg^−1^ DM at 4,000 m; however, F4500 and F5000 showed lower CP concentration and the lowest WSC concentration among all fresh grasses. This might be attributed to the advanced lignification of plant cell walls in high-elevation regions. The *K. pygmaea* in this study were harvested at narrower harvest window within 15 days instead of consistent growth stage, and the vegetative growth of *K. pygmaea* in high-elevation regions might be premature termination compared with that of the low-elevation regions. Han and Ren (2015) reported that the end of the growth season advanced 0.6 day for every 200 m higher elevations. Thus, more CP and WSC in F4500 and F5000 were embedded in lignified cell walls than other grasses.

F3000 and F5000 showed the highest and lowest microbial populations, respectively. This might be attributed to their higher and lower moistures than *K. pygmaea* harvested from other altitudes. It is well known that decreasing moisture (relative humidity) creates a less hospitable environment for microorganisms to grow, although different types of fungus or bacteria require different amounts of water (in vapor form) to reproduce and grow (Cabral, 2010). Microbes that have the ability to survive in extremely cold, strong ultraviolet rays, and hypoxia conditions are rare, Dong et al. (2010) also reported that bacterial isolates obtained from Tibetan lakes were either phylogenetically or physiologically unique. The lowest numbers of yeast, Enterobacteriaceae, and total bacteria in F5000 among all fresh *K. pygmaea* might be attributed to the harsh environment, which is characterized by low temperature, strong ultraviolet rays, and hypoxia.

### Altitudinal Distribution Patterns of Microbial Community in Fresh *K. pygmaea*

The decrease in the phyllosphere fungal diversity might be related to the increased physiological challenges with the increasing altitude, resulting in fewer fungal species survived in the highest elevations. However, the phyllosphere bacterial diversity increased with the increasing altitude. It was well known that bacteria and archaea had higher adaptation ability to extreme environments than fungi because of the lower complexity of prokaryotic cells compared to eukaryotic fungi (Gostinčar et al., 2022). Bacterial and fungal richness showed a unimodal distribution, with peak abundance at F4000 and F3000, respectively. This is consistent with the pattern of plant richness at large spatial scales, which showed a unimodal relationship with altitude, with the peaked richness in the middle elevations (Oommen and Shanker, 2005).

Plant phyllosphere harbor a diverse variety of microorganisms, which have been shown to play an important role in the adaptation of the plant host to different environmental stressors by enhancing tolerance to heat, cold, drought, UV radiation, and salinity (Al Ashhab et al., 2021). Plants in cold environments harbor complex, host-specific, and cold-adapted microbial communities, which may play a key functional roles in plant growth and survival under cold conditions. In this study, the dominant epiphytic bacteria in fresh *K. pygmaea* are *Enterobacter, Exiguobacterium, Curtobacterium*, and *Pseudomonas*, which were usually isolated from cold environment. Marian et al. (2022) reviewed that *Pseudomonas* spp. isolated from cold environments could be used to promote plant growth under cold stress, whereas other microorganisms isolated from cold environments (e.g., *Enterobacter, Exiguobacterium*, and *Pseudomonas*) promoted plant growth under normal conditions (16–25°C), although their effect on cold-stressed plants was not yet investigated. The dominant epiphytic bacterial genera in fresh *K. pygmaea* also were difference from that of fresh corn cultivated in low-altitude region of Southwestern China, which was dominated by *Lactobacillus, Acetobacter, Weissella, Pseudomonas, Acinetobacter*, and *Burkholderia* (Guan et al., 2018).

*Streptococcus* is detected in *K. pygmaea* harvested from 4,000 m a.s.l. Fan et al. (2020) reported that the RA of *Streptococcus* in the rumen of yaks increased with altitude and proposed that these microorganisms might adapt well to low-temperature and low-oxygen environments. *Sphingomonas* are observed in *K. pygmaea* harvested from higher elevations (above 4,000 m a.s.l.). Some species of *Sphingomonas* can tolerate extreme cold conditions and be found in high-altitude and low-temperature environments of the Indian Himalayas (Dasauni and Nailwal, 2020) because they can produce many antioxidative enzymes to reduce the damage caused by reactive oxygen species (Luo et al., 2020). *Acidiphilium* and *Arthrobacter* were only detected in the highest elevation of 5,000 m a.s.l., where they are characterized by extreme environments. Li L. et al. (2020) performed the comparative genomic analysis on *Acidiphilium* to address the evolutionary history of the genus *Acidiphilium* and proposed that *Acidiphilium* originated in mild conditions and could adapt to extreme environments after the acquisition of many essential functions. Members of *Arthrobacter* are commonly identified in Antarctic soil communities. Dsouza et al. (2015) reported that the genomes of the seven Antarctic *Arthrobacter* isolates contained several features that may be beneficial for growth and survival in the Antarctic soil environment. Ge et al. (2020) also isolated four unknown strains belonging to the genus *Arthrobacter* from plateau wildlife on the Tibetan Plateau.

For the fungi, *Pyrenocchaetopsis* are present in fresh *K. pygmaea* from elevations below 4,000 m a.s.l., and its RA decreased with the increasing elevation. *Pyrenochaetopsis* was one of the rhizospheric soil fungal genera in low-altitude sites rather than high- and medium-altitude sites (Jamil et al., 2020). *Protomyces* and *Leucosporidium* are present in fresh *K. pygmaea* from elevations below 4,000 m a.s.l. before ensiling, and its RA increased with the increasing elevation. *Rhodotorula* and *Saitozyma* were found in all fresh *K. pygmaea* except F5000 with the highest RA in F4000. *Rhodotorula himalayensis* sp. has been isolated from Roopkund Lake of the Himalayan mountain ranges (Shivaji et al., 2008). *Saitozyma pseudoflava* also has been isolated from phylloplane in Tibet, China (Li A. H. et al., 2020). In the study by Touchette et al. (2022), *Rhodotorula* JG1 was used as a model yeast to determine its adaptations to cold temperatures and found that *Rhodotorula frigidialcoholis* adapted to cold temperatures in the Antarctic dry valley permafrost through a variety of mechanisms, including increasing expression of the PPP genes, increasing the production of carotenoids, sphingolipids, unsaturated fatty acid, and exopolysaccharides while coupled with a reduction in expression of growth, transcriptional and translational machinery genes. *Didymella, Ariculibuller, Zymoseptoria*, and *Sampaiozyma* are found in *K. pygmaea* from lower elevations (below 3,000 m a.s.l.); however, they are absent in higher elevations. *Didymella* and *Zymoseptoria* were also detected in fresh corn (Benjamim Da Silva et al., 2021; Kung et al., 2021). The exclusive presence of *Unclassified_f_Helotiaceae* and *Unclassified_p_Ascomycota* in F4500 and F5000 may be explained by a combination of geographic isolation and unique environmental conditions on the Tibetan Plateau. This indicated that high-altitude Tibetan Plateau harbor its unique assemblage of fungi, which could well be adapted to the extreme conditions.

### Correlations Between Environmental Parameters and Microbial Community in Fresh *K. pygmaea*

Climate is one of the major factors that shape microbial communities in the nature. The variations in phyllosphere bacterial community were related to environmental and biotic factors (Al Ashhab et al., 2021). Physiological profiles of the crop concerning abiotic factors affect the availability of nutrients, water, and a wide range of secondary metabolites on the leaf surface, which further and therefore significantly affect the epiphytic microbial communities (Liu et al., 2020). Altitude is strongly influenced by the epiphytic bacterial and fungal community composition. In this study, relative humidity, temperature, and altitude have a strong effect explaining 41.56 and 15.57% in axes 1 and 2, respectively, accounting for the total variance in bacterial community composition (Figure 4A). Beattie (2002) also found that the microbial communities of leaves were significantly affected by leaves' moisture.

In the fungal community, relative humidity, temperature, and precipitation explained 26.04 and 19.37% in CCA axes 1 and 2, respectively, of the total variations of fungal community (Figure 4B). Temperature and measures of moisture availability, including relative humidity and precipitation, affected the fungal community composition. Faticov et al. (2021) have shown that the fungal community composition changed along the elevational gradient and was also affected by precipitation.

### The Fermentation Profiles of *K. pygmaea* Along the Elevation Gradient

Ensiling without any inoculants or starter cultures is a natural and spontaneous fermentative process depending on the epiphytic microorganism. In this study, all silage pH values are above 5.0, indicating that all *K. pygmaea* underwent a weak LA-fermentation. Kung et al. (2018) reviewed that the ratio of LA/AA is a qualitative indicator of silage fermentation, and quality silage usually has a ratio of LA/AA about 2.5–3.0. In this study, the LA/AA of all silages ranged from 0.56 to 2.19, confirming the poor fermentation of *K. pygmaea* silages. Especially, S5000 silages showed the lowest LA/AA (0.56) and were dominated by the bacterial family of Enterobacteriaceae (76.59%) (Avila and Carvalho, 2020). The high AA concentrations of *K. pygmaea* silages in this study was attributed to the flourish of *Enterobacteria*, metabolizing fermentable substrates into AA (Heron et al., 1993). Most of the *Enterobacteria* are undesirable for silage fermentation because they compete with LAB for sugars at the beginning of ensiling; however, *Enterobacteria* are generally sensitive to low pH, and their counts in silage usually decline sharply when the pH falls below 4.5 (McGarvey et al., 2013). In this study, the slow decline of pH favors the proliferation of *Enterobacteria*.

The highest pH in S5000 silages was related to its highest DM, which could not provide sufficient moisture to support the growth of bacteria, restricting the silage fermentation. In addition, the high DM contents makes forage difficult to be compacted to generate anaerobic environment for ensiling fermentation, resulting in the enrichment of aerobic yeast, such as *Rhodotorula*, which are non-fermentative and strictly aerobic yeast (Hatoum et al., 2012). In this study, *Rhodotorula* became the dominant fungal genus in S5000 after 60 days of ensiling. In contrast, S3000 silages showed the lowest pH and highest LA concentration among all silages, which are related to their highest LAB numbers and lowest DM contents in fresh *K. pygmaea*. Proper moisture at harvest is essential to getting a good pack and fermentation (Borreani et al., 2018). In this study, although *Lactobacillus* dominated the S4000 after 60 days of ensiling, S4000 still exhibited poor fermentation quality as well as other four regions. The lack of high-efficiency LAB and fermentable substrates might contribute to the restricted fermentation of *K. pygmaea*.

The flourish of *Enterobacteria* partially contributed to the prevalence of ammonia N in silage because *Enterobacteria* had a proteolytic activity to produce ammonia and biogenic amines (Driehuis et al., 2018). In this study, the higher ammonia N concentrations in S3000 and S4000 than other silages were consistent with the detectable butyric acid in S3000 and S4000. Accordingly, poorly preserved silage is obtained with high contents of butyric acid and ammonia N, which is associated with clostridial activity (Buxton and Muck, 2003). The lowest ammonia N concentration in S5000 was related to its highest DM among all silages. The chances of a clostridial fermentation can be minimized by ensiling forages above 35% DM because clostridia are intolerant of high osmotic pressure (Kung et al., 2018).

In the fresh *K. pygmaea* before ensiling, the phyllosphere bacterial community exhibited regular altitudinal distribution pattern; however, the dominant bacterial genera in silages were absolutely different among five elevations after 60 days of ensiling. Zi et al. (2021) reported that environmental factors affected the epiphytic bacterial community of forages, which have a greater impact on the initial stage of silage fermentation, whereas their effects on the microbial communities were reduced with the process of ensiling. In addition, they suggested that geographical factors and their effect on the silage microbiome should be considered to make provisions to ensure high-quality silage production (Zi et al., 2021). In this study, the complex epiphytic microbial composition contributed to the poor fermentation quality of *K. pygmaea* silages. Thus, it is concluded that inoculating LAB to *K. pygmaea* on the Tibetan plateau before ensiling is highly recommended.

The major fungal genera identified in fresh *K. pygmaea* were *Pyrenochaetopsis, Auriculibuller, Saitozyma, Rhodotorula*, and *Unclassified_f* _*Helotiaceae*. After 60 days of ensiling, the fungal composition significantly changed, and *Schizophyllum, Phodotorula, Rhodotorula, Candida*, and *Issatchenkia* became the most dominant fungal genera in silages. Most molds do not tolerate at low pH and low-oxygen concentrations; nevertheless, yeasts can remain active due to their tolerance to low pH and their ability to ferment sugars to ethanol, allowing them to survive during the ensiling process (Avila and Carvalho, 2020). There are significant differences in fungal community among *K. pygmaea* 60-day silages from five regions, which may have been influenced by the initial fungal community composition of fresh material (Romero et al., 2021).

## Conclusion

The altitude is a crucial factor that shapes the phyllosphere bacterial and fungal communities of *K. pygmaea*, which has unique bacterial and fungal community composition in high elevation gradient of the Tibetan Plateau. The natural ensiling of *K. pygmaea* underwent a weak LA fermentation, indicated by high pH above 5.0 and low LA/AA. Thus, considering the complex epiphytic microbial composition in fresh *K. pygmaea*, inoculating LAB to *K. pygmaea* before ensiling is highly recommended on the Tibetan Plateau.

## Data Availability Statement

The original contributions presented in the study are included in the article/Supplementary Material, further inquiries can be directed to the corresponding author/s.

## Author Contributions

XYu: conceiving the idea, designing the experiment, and writing—review and editing. XYa: sampling and analysis, data analysis, writing–original draft, and writing—review and editing. YB: formal analysis, methodology, and writing—review and editing. AG: writing—review and English editing. TS, WenkW, PM, and WenbW: sampling, analysis, and editing. All co-authors participated in discussions and revised the manuscript. All authors contributed to the article and approved the submitted version.

## Funding

This research was supported by the National Natural Science Foundation of China (31960355), Integration and Demonstration of Crop Straw Comprehensive Utilization Technology in Tibet (XZ201901NB07), and State Key Laboratory of Barley and Yak Germplasm Resources and Genetics Improvement (Tibet Academy of Agricultural and Animal Husbandry Sciences) (XZNKY-2019-C-007K09).

## Conflict of Interest

The authors declare that the research was conducted in the absence of any commercial or financial relationships that could be construed as a potential conflict of interest.

## Publisher's Note

All claims expressed in this article are solely those of the authors and do not necessarily represent those of their affiliated organizations, or those of the publisher, the editors and the reviewers. Any product that may be evaluated in this article, or claim that may be made by its manufacturer, is not guaranteed or endorsed by the publisher.
