# Supplementary material for: Altitudinal Distribution Patterns of Phyllosphere Microbial Communities and Their Contribution to Silage Fermentation of Kobresia pygmaea Along the Elevation Gradient on the Tibetan Plateau
Source: Front Microbiol. 2022 May 24;13:874582. doi: 10.3389/fmicb.2022.874582 (PMC9173736; doi:10.3389/fmicb.2022.874582)
Supplement: Supplementary file 1 [file Data_Sheet_1.docx]

Table S1 Description of the sampling sites

| Item | Atmospheric  Pressure (Pa) | Average  temp (°C) | Relative humidity(%) | Average precip(mm) | Relative wind  speed(m/s) | Latitude | Longitude |
| --- | --- | --- | --- | --- | --- | --- | --- |
| Elevation (m a.s.l.) |  |  |  |  |  |  |  |
| 2500 | 732.2 | 11.34 | 67.89 | 51.22 | 1.52 | N29°54′14″ | E95°30′3″ |
| 3000 | 708.9 | 10.54 | 62.03 | 51.54 | 1.81 | N29°33′58″ | E94°30′16″ |
| 4000 | 713.8 | 10.66 | 66.84 | 50.52 | 1.20 | N29°26′1″ | E94°36′25″ |
| 4500 | 708.9 | 10.54 | 62.03 | 51.55 | 1.81 | N29°37′20″ | E94°39′52″ |
| 5000 | 1340 | 8.48 | 45.00 | 63.57 | 2.56 | N29°49′39″ | E92°20′37″ |

Table S2 The effect of environment factors on bacterial community of *Kobresia pygmaea* before ensiling along the elevation gradient on the Tibetan Plateau

| Item | RDA1 | RDA2 | r2 | P-values |
| --- | --- | --- | --- | --- |
| Altitudes | 0.9786 | -0.2056 | 0.876 | 0.001 |
| Temperature(°C) | -0.8301 | 0.5577 | 0.4819 | 0.018 |
| Relative humidity (%) | -0.664 | 0.7477 | 0.5238 | 0.009 |
| Crude protein | 0.6271 | 0.779 | 0.8079 | 0.001 |
| Water-soluble carbohydrate | -0.6539 | 0.7566 | 0.3312 | 0.087 |
| Dry matter | 0.8023 | -0.5969 | 0.6791 | 0.002 |

Table S3 The effect of environment factors on the axes bacterial community of *Kobresia pygmaea* and after ensiling along the elevation gradient on the Tibetan Plateau

| Item | RDA1 | RDA2 | r2 | P-values |
| --- | --- | --- | --- | --- |
| Temperature(°C) | 0.2563 | -0.9666 | 0.8337 | 0.004 |
| Relative humidity (%) | 0.4461 | -0.895 | 0.8231 | 0.003 |
| Average precip (mm) | -0.3652 | 0.9309 | 0.7146 | 0.005 |
| Neutral detergent fiber | -0.9987 | -0.0501 | 0.4146 | 0.047 |

Table S4 The effect of environment factors on the axes fungal community of *Kobresia pygmaea* before ensiling along the elevation gradient on the Tibetan Plateau

| Item | CCA1 | CCA2 | r2 | P-values |
| --- | --- | --- | --- | --- |
| Altitudes | 0.6562 | -0.7546 | 0.9649 | 0.001 |
| Temperature(°C) | -0.9853 | 0.171 | 0.9456 | 0.001 |
| Relative humidity (%) | -0.9956 | 0.0932 | 0.9334 | 0.001 |
| Average precip (mm) | 1 | 0.0005 | 0.9935 | 0.002 |
| Dry matter | 0.9695 | -0.245 | 0.9827 | 0.001 |

Table S5 The effect of environment factors on the axes fungal community of *Kobresia pygmaea* after ensiling along the elevation gradient on the Tibetan Plateau

| Item | CCA1 | CCA2 | r2 | P-values |
| --- | --- | --- | --- | --- |
| Altitudes | 0.945 | -0.327 | 0.5095 | 0.007 |
| Temperature(°C) | -0.6747 | 0.7381 | 0.881 | 0.002 |
| Relative humidity (%) | -0.6515 | 0.7586 | 0.8413 | 0.004 |
| Average precip (mm) | 0.7382 | -0.6746 | 0.9193 | 0.003 |
| Neutral detergent fiber | -0.755 | -0.6557 | 0.5744 | 0.004 |
| Water-soluble carbohydrate | 0.7959 | -0.6055 | 0.8531 | 0.001 |
| Dry matter | 0.8728 | -0.4881 | 0.8991 | 0.001 |


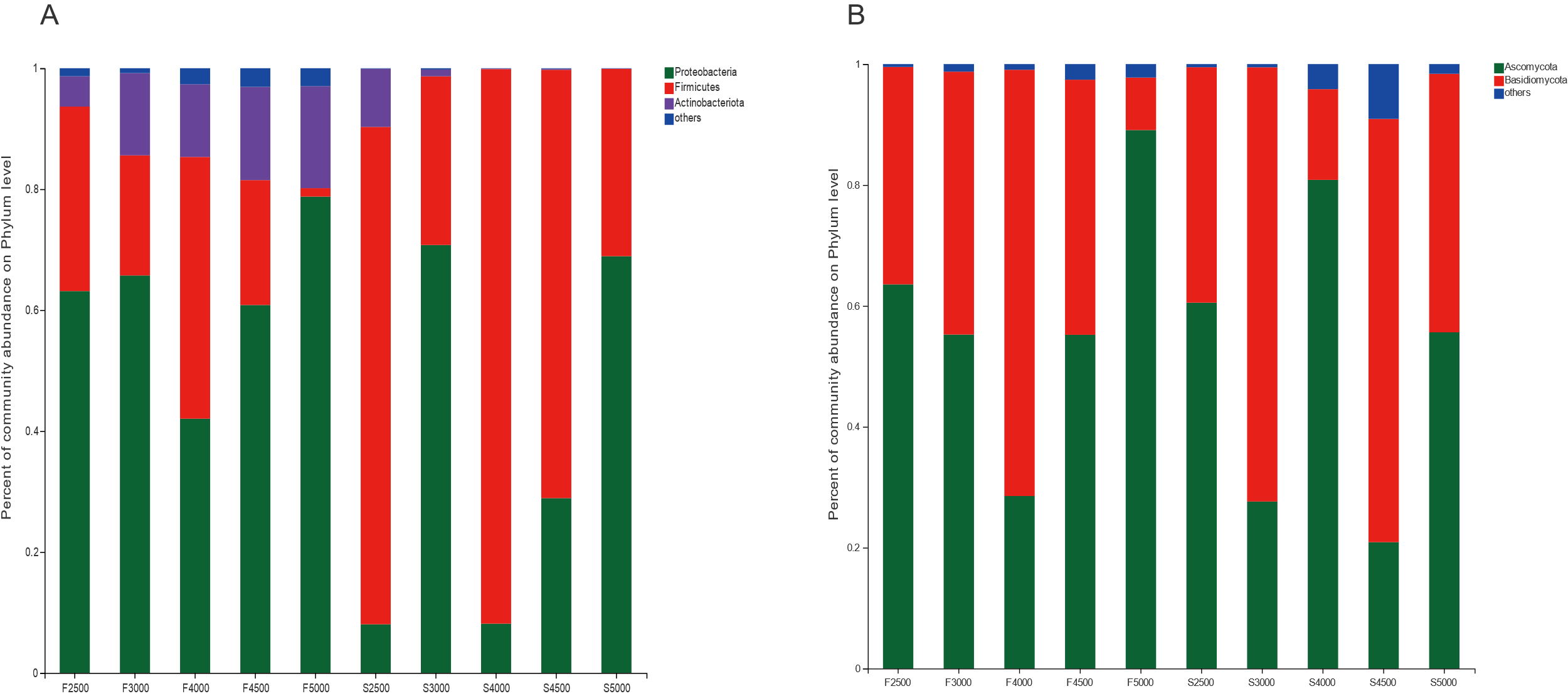


Figure S1. The bacterial (A) and fungal (B) community on phylum levels of *Kobresia pygmaea* along the elevation gradient on the Tibetan Plateau. F2500, F3000, F4000, F4500, F5000 represent samples of fresh *Kobresia pygmaea* at corresponding altitude gradients. S2500, S3000, S4000, S4500, S5000 represent samples of *Kobresia pygmaea* after 60 d of ensiling at corresponding altitude gradients.
